# Genomic characterization of an NDM-9-producing *Acinetobacter baumannii* clinical isolate and role of Glu152Lys substitution in the enhanced cefiderocol hydrolysis of NDM-9

**DOI:** 10.3389/fmicb.2023.1253160

**Published:** 2023-08-28

**Authors:** Susie Gaillot, Saoussen Oueslati, Jean-Baptiste Vuillemenot, Maxime Bour, Bogdan I. Iorga, Pauline Triponney, Patrick Plésiat, Rémy A. Bonnin, Thierry Naas, Katy Jeannot, Anaïs Potron

**Affiliations:** ^1^Université de Franche-Comté, UMR CNRS 6249 Chrono-Environnement, Besançon, France; ^2^Université Paris-Saclay, Equipe INSERM ReSIST, Faculté de Médecine, Le Kremlin-Bicêtre, France; ^3^Laboratoire Associé du Centre National de Référence de la Résistance aux Antibiotiques, Centre Hospitalier Universitaire de Besançon, Besançon, France; ^4^Université Paris-Saclay, CNRS Institut de Chimie des Substances Naturelles, Gif-sur-Yvette, France; ^5^Laboratoire Associé du Centre National de Référence de la Résistance aux Antibiotiques: Entérobactéries Résistantes aux Carbapénèmes, Le Kremlin-Bicêtre, France

**Keywords:** *Acinetobacter baumannii*, cefiderocol, carbapenemases, NDM, resistance

## Abstract

Here, we characterized the first French NDM-9-producing *Acinetobacter baumannii* isolate. *A. baumannii* 13A297, which belonged to the ST^Pas^25 (international clone IC7), was highly resistant to β-lactams including cefiderocol (MIC >32 mg/L). Whole genome sequencing (WGS) using both Illumina and Oxford Nanopore technologies revealed a 166-kb non-conjugative plasmid harboring a *bla*_NDM-9_ gene embedded in a Tn*125* composite transposon. Complementation of *E. coli* DH5α and *A. baumannii* CIP70.10 strains with the pABEC plasmid carrying the *bla*_NDM-1_ or *bla*_NDM-9_ gene, respectively, resulted in a significant increase in cefiderocol MIC values (16 to >256-fold), particularly in the NDM-9 transformants. Interestingly, steady-state kinetic parameters, measured using purified NDM-1 and NDM-9 (Glu152Lys) enzymes, revealed that the affinity for cefiderocol was 3-fold higher for NDM-9 (*K*_m_ = 53 μM) than for NDM-1 (*K*_m_ = 161 μM), leading to a 2-fold increase in catalytic efficiency for NDM-9 (0.13 and 0.069 μM^−1^.s^−1^, for NDM-9 and NDM-1, respectively). Finally, we showed by molecular docking experiments that the residue 152 of NDM-like enzymes plays a key role in cefiderocol binding and resistance, by allowing a strong ionic interaction between the Lys152 residue of NDM-9 with both the Asp223 residue of NDM-9 and the carboxylate group of the R1 substituent of cefiderocol.

## 1. Introduction

*Acinetobacter baumannii* is definitely one of the most successful pathogens responsible for healthcare-associated infections. Its propensity to acquire and accumulate antibiotic resistance genes has led to its classification by the WHO as one of the 12 top priority resistant bacteria (Tacconelli et al., 2018). Carbapenem resistance in *A. baumannii* (CRAB) is a serious threat to public health, and prevalence of CRAB, which varies widely between countries, has been increasing worldwide [Antimicrobial Resistance in the EU/EEA (EARS-Net), 2022]. Resistance of *A. baumannii* strains to carbapenems most commonly results from the acquisition of carbapenem-hydrolyzing class D β-lactamases (CHDL), belonging to the OXA-23-like, OXA-24/40-like, OXA-58-like, OXA-143-like and OXA-235-like groups (Rodríguez et al., 2018; Ramirez et al., 2020). However, the incidence of NDM-like class B (MBL) β-lactamases has steadily grown recently (Ramirez et al., 2020). Of the 55 NDM variants listed in the Beta-Lactamase DataBase (Naas et al., 2017), NDM-1 is the most frequently NDM-type carbapenemase identified in CRAB but strains producing other variants, such as NDM-2, NDM-5, NDM-6 or more recently NDM-42, have been sporadically reported (Kaase et al., 2011; Khalid et al., 2020; Xanthopoulou et al., 2020; Quiñones Pérez et al., 2022). While the *bla*_NDM-9_ variant was very rarely detected in *A. baumannii* (Poirel et al., 2021; Le Terrier et al., 2023), NDM-9 has been reported more frequently in *Enterobacterales* (*K. pneumoniae* and *E. coli*), especially in East Asia (South Korea, Taiwan, China and French Polynesia) and sporadically in Europe (Switzerland, Italy) (Wang et al., 2014; Di et al., 2017; Nüesch-Inderbinen et al., 2018; Lin et al., 2019; Falcone et al., 2020; Oueslati et al., 2021). More recently, it has been shown that the production of the NDM-9 enzyme in *Enterobacterales* and in an *A. baumannii* strain impairs the activity of new combinations of β-lactam-β-lactamase inhibitors (ceftazidime/avibactam, imipenem/relebactam, and meropenem/vaborbactam), including one under development (cefepime/taniborbactam) (Le Terrier et al., 2023). The most worrying threat of NDM-like producing *A. baumannii* is the inconsistent efficacy of cefiderocol, a recently commercialized molecule that is considered a last-resort antibiotic in the fight against CRAB. Even though some NDM-producing *A. baumannii* strains are fully susceptible to cefiderocol (Yamano, 2019; Mushtaq et al., 2020), cefiderocol MICs tend to be higher for NDM-producing organisms as compared to strains expressing other carbapenemases (Yamano, 2019; Mushtaq et al., 2020; Poirel et al., 2021). Undoubtedly, additional factors likely contribute to the high cefiderocol resistance of some bacteria (Yamano, 2019; Kohira et al., 2020; Simner and Patel, 2020), and in *A. baumannii*, downregulation or absence of specific siderophore receptors (PirA and PiuA) and alterations of PBP3 have been suggested (Malik et al., 2020). In this study, we report the genetic environment of the *bla*_NDM-9_ gene identified in a clinical strain of *A. baumannii*, and the comparison of steady-state kinetic parameters between the NDM-9 and NDM-1 purified enzymes for cefiderocol.

## 2. Materials and methods

### 2.1. Strains used in the study

*Acinetobacter baumannii* clinical strain 13A297 was isolated in 2013 from a urine sample collected from a patient hospitalized in France. Species identification was performed by MALDI-TOF mass spectrometry and confirmed by whole genome sequencing. Clinical *A. baumannii* strains 19A2597 and 13A297, producing the NDM-1 and NDM-9 β-lactamase, respectively, were selected for cloning of the respective genes into the pABEC vector and then transferred into *E. coli* DH5α and *A. baumannii* CIP70.10 (Vuillemenot et al., 2022). Mating out assays were carried out using the rifampicin-resistant BM4547 strain derived from CIP70.10 as the recipient strain (Potron et al., 2014). The transconjugants were selected on Mueller-Hinton agar plates supplemented with ticarcillin (100 mg/L) and rifampicin (100 mg/L). *E. coli* BL21 (DE3) (Novagen, Fontenay-sous-Bois, France) was used for overproduction of NDM-1 and NDM-9 enzymes.

### 2.2. Susceptibility testing

MICs of β-lactams (ticarcillin, piperacillin-tazobactam, ceftazidime, cefepime, imipenem, meropenem), ciprofloxacin, trimethoprim-sulfamethoxazole, amikacin, tigecycline, minocycline and colistin were determined by the broth microdilution method in cation-adjusted Mueller-Hinton broth (MHB), by using customized microplates (ThermoFisher, Illkirch-Graffenstaden, France). Cefiderocol MICs were determined using an Iron-Depleted and Cation-Adjusted Mueller-Hinton Broth (ID-CAMHB) and a titrated powder of cefiderocol (MedChem Express) according to the recommendations of the Clinical and Laboratory Standards Institute (2020). MIC values were interpreted according to the current EUCAST or CLSI (for minocycline and tigecycline) clinical breakpoints, except for cefiderocol, for which the EUCAST pharmacokinetic-pharmacodynamic (PK-PD) breakpoint (S ≤ 2; R > 2 mg/L) was applied [Clinical and Laboratory Standards Institute, 2020; The European Committee on Antimicrobial Susceptibility Testing (EUCAST), 2021].

### 2.3. Genomic data

Total DNA of strains 13A297 and 19A2597 was purified (NucleoSpin Microbial DNA, Macherey-Nagel, Germany), and then quantified spectrophotometrically (NanoDrop, Thermo Fisher Scientific, France). Preparation of DNA libraries and whole-genome sequencing experiments were outsourced to services using Illumina platforms generating paired-end reads (P2M platform, Institut Pasteur, Paris, France). In addition, long-reads sequencing using the Oxford Nanopore technology was performed for the strain 13A297 (MinION, R9.4.1 flow cell). Short and long reads were assembled as hybrid using SPAdes v.3.15.5 and Trycycler v.0.5.0. Quality control of reads, assembly, and genome annotation were carried out using FastQC v.0.11.9, QUAST v.5.0.2, and RASTtk on the PATRIC 3.6.9 platform. Antimicrobial resistance genes (AMR) were identified by the National Reference Center for Antimicrobial Resistance (NRC-AR) pipelines and databases. The sequence type (ST) of isolates was determined according to the MLST schemes available at PubMLST.^1^

### 2.4. Molecular experiments

From genomic DNA extracted using the QIAamp DNA mini kit (Qiagen, Courtaboeuf, France), the *bla*_NDM-1_ and *bla*_NDM-9_ genes were amplified with the specific primers NDM_fwd (5’-CAAGCTTGGTACCGAGCTCGTGGCTTTTGAAACTGTCGC-3′) and NDM_rev (5’-ACTGGCGGCCGTTACTAGTGTCGAGGTCAGGATAGGGG-3′) and subsequently cloned into the shuttle vector pABEC using the NEBuilder Hifi assembly kit according to the manufacturer’s recommendations (New England Biolabs, Evry, France) (Vuillemenot et al., 2022).

For the production of NDM-1 and NDM-9 proteins, the complete coding sequences of the β-lactamase genes were amplified by PCR using the specific primers NdeI-NDM_29-270_ (5′-AAAAACATATGGGTGAAATCCGCCCGACG-3′) and NDM-XhoI_delta STOP_ (5′-AAAAACTCGAGGCGCAGCTTGTCGGCCAT-3′). The amplicons and the plasmid pET41b were digested with the restriction enzymes NdeI and XhoI before ligation. The recombinant plasmids pET41b-NDM-1 and pET41b-NDM-9 were transferred by transformation into *E. coli* BL21 (DE3).

### 2.5. Protein purification

Two liters of brain heart infusion broth medium containing 30 mg/L kanamycin were inoculated with 2 mL overnight cultures of *E. coli* strains BL21 (DE3) pET41b-NDM-1 and of *E. coli* BL21 (DE3) pET41b-NDM-9. Production of recombinant proteins harboring the His tag was induced overnight at 22°C with 0.2 mM IPTG. Then, the cultures were centrifuged at 6,000 g for 15 min and the pellets were resuspended with the binding buffer (10 mM imidazole, 25 mM sodium phosphate pH 7.4 and 300 mM NaCl). Bacterial cells were lysed by sonication and the pellet was removed by two consecutive centrifugation steps at 10,000 g for 1 h at 4°C; the supernatant was then centrifuged at 96,000 g for 1 h at 4°C. The soluble fractions were filtered, passed through a HisTrap^TM^ HP column (GE Healthcare) and proteins were eluted with the elution buffer (500 mM imidazole, 25 mM sodium phosphate pH 7.4 and 300 mM NaCl). Finally, gel filtration was performed with 50 mM sodium phosphate buffer pH 7 and 150 mM NaCl with a Superdex 75 column (GE Healthcare). The fractions were pooled according to purity by SDS–PAGE, dialyzed in 100 mM sodium phosphate pH 7, 50 μM ZnSO_4_, and concentrated using Vivaspin columns. The concentration was determined by measuring the OD at 280 nm and with the extinction coefficients obtained from the ProtParamtool (Swiss Institute of Bioinformatics online resource portal).

### 2.6. Steady-state kinetic determinations

Kinetic parameters were determined using purified NDM-1 and NDM-9 β-lactamases in 20 mM Hepes pH 7.5 supplemented with 50 μM ZnSO_4_. The *k*_cat_ and *K*_m_ values were determined by analyzing β-lactam hydrolysis under initial-rate conditions with an ULTROSPEC 2000 UV spectrophotometer with the SWIFT II software (GE Healthcare, Velizy-Villacoublay, France) and the Eadie-Hofstee linearization of the Michaelis–Menten equation.

### 2.7. Molecular modeling

Three-dimensional structure of NDM-9 was generated by *in silico* mutagenesis from the structure of NDM-1 (PDB code 4RL0) (Feng et al., 2014) using UCSF Chimera (version 1.14) (Pettersen et al., 2004). Molecular docking of cefiderocol (open form) into the active sites of both NDM-1 and NDM-9 was performed using CCDC Gold (part of CSD 2021 software) (Verdonk et al., 2003) with the GoldScore scoring function and the binding site defined as a sphere with 20 Å radius around Zn301.

### 2.8. Nucleotide sequence accession number

The whole genome sequences generated in the study have been submitted to the Genbank nucleotide sequence database under the accession number PRJNA989410.

## 3. Results and discussion

### 3.1. Phenotypic and genomic characterization of *Acinetobacter baumannii* 13A297 clinical isolate

*Acinetobacter baumannii* 13A297 strain was resistant to all β-lactams, including cefiderocol (MIC >32 mg/L), aminoglycosides, fluoroquinolones, trimethoprim-sulfamethoxazole and remained susceptible to colistin and tetracyclines (Table 1). WGS sequencing of *A. baumannii* 13A297 revealed a genome of 4,229,816 bp with a mean coverage of 280 X. Analysis of WGS data revealed that this isolate possessed the naturally-occurring *bla*_OXA-64_ and *bla*_ADC-26_ genes. Its resistance profile to β-lactams was the result of acquisition of the carbapenemase NDM-9, that differs from NDM-1 by a single amino-acid substitution (Glu152Lys). NDM-9 was first reported in a clinical *Klebsiella pneumoniae* isolate from China in 2013 (Wang et al., 2014). This enzyme was also identified in a *mcr-1*-harboring *Escherichia coli* strain, in three environmental *Klebsiella variicola* isolates in South Korea (Yao et al., 2016; Di et al., 2017) and in a *K. pneumoniae* strain isolated from wastewater in Switzerland in 2015 (Nüesch-Inderbinen et al., 2018). Since then, NDM-9 was sporadically detected in clinical, environmental or animal strains belonging to various *Enterobacterales* species including *Salmonella* sp. in Asia and Europe (Italy), but also in Africa (Tunisia) and in French Polynesia (Falcone et al., 2020; Li et al., 2021; Oueslati et al., 2021; Song et al., 2022; Sun et al., 2023; Zouaoui et al., 2023). MLST assigned *A. baumannii* 13A297 strain to ST^Pas^25, and ST^Ox^2280, according to the MLST Pasteur and Oxford schemes, respectively. In addition to the globally disseminated multidrug-resistant ST^Pas^1 and ST^Pas^2, ST^Pas^25, which belongs to international clone 7 (IC7), has emerged worldwide as epidemic, endemic or sporadic cases in the last two decades (Sahl et al., 2015; Rodríguez et al., 2018; Rafei et al., 2022). A hallmark of this clone is its evolution toward multidrug resistance and a high capacity for biofilm formation (Rafei et al., 2022). In addition, it has been associated with high mortality rates among infected patients (da Silva et al., 2018).

**Table 1:** MICs (mg/L) of β–lactams and other antibiotics for *A. baumannii* 13A297, *A. baumannii* CIP70.10 (pABEC), *A. baumannii* CIP70.10 (pABEC-NDM-1), *A. baumannii* CIP70.10 (pABEC-NDM-9), *E. coli* DH5α (pABEC), *E. coli* DH5α (pABEC-NDM-1), and *E. coli* DH5α (pABEC-NDM-9).

Antibiotic	*A. baumannii* 13A297	*E. coli* DH5α			*A. baumannii* CIP70.10		
		pABEC-NDM-1	pABEC-NDM-9	pABEC	pABEC-NDM-1	pABEC-NDM-9	pABEC
Ticarcillin	>512	>512	>512	2	>512	>512	32
Piperacillin-tazobactam^a^	64	>64	>64	≤2	>64	>64	4
Ceftazidime	>128	>32	>32	<0.5	>32	>32	4
Cefepime	>128	32	>32	<0.5	>32	>32	2
Imipenem	64	>16	>16	<0.5	>16	>16	0.5
Meropenem	>64	16	>16	<0.12	>16	>16	0.5
Cefiderocol^b^	**>32**	2	**8**	<0.03	**4**	**>32**	0.25
Amikacin	128	≤1	≤1	≤1	≤1	≤1	≤1
Tigecycline	0.5	≤0.25	≤0.25	≤0.25	≤0.25	≤0.25	≤0.25
Minocycline	≤0.5	≤0.5	≤0.5	≤0.5	≤0.5	≤0.5	≤0.5
Trimethoprim-sulfamethoxazole	>8	≤1/19	≤1/19	≤1/19	≤1/19	≤1/19	≤1/19
Ciprofloxacin	>16	≤0.25	≤0.25	≤0.25	≤0.25	≤0.25	≤0.25
Colistin	1	≤0.25	≤0.25	≤0.25	1	1	1

^a^Tazobactam 4 mg/L.

^b^The values higher than that of the EUCAST clinical PK-PD cefiderocol concentration (> 2 μg/mL) are indicated in bold.

### 3.2. Genetic support and environment of *bla*_NDM-9_ gene

**Figure 1:**
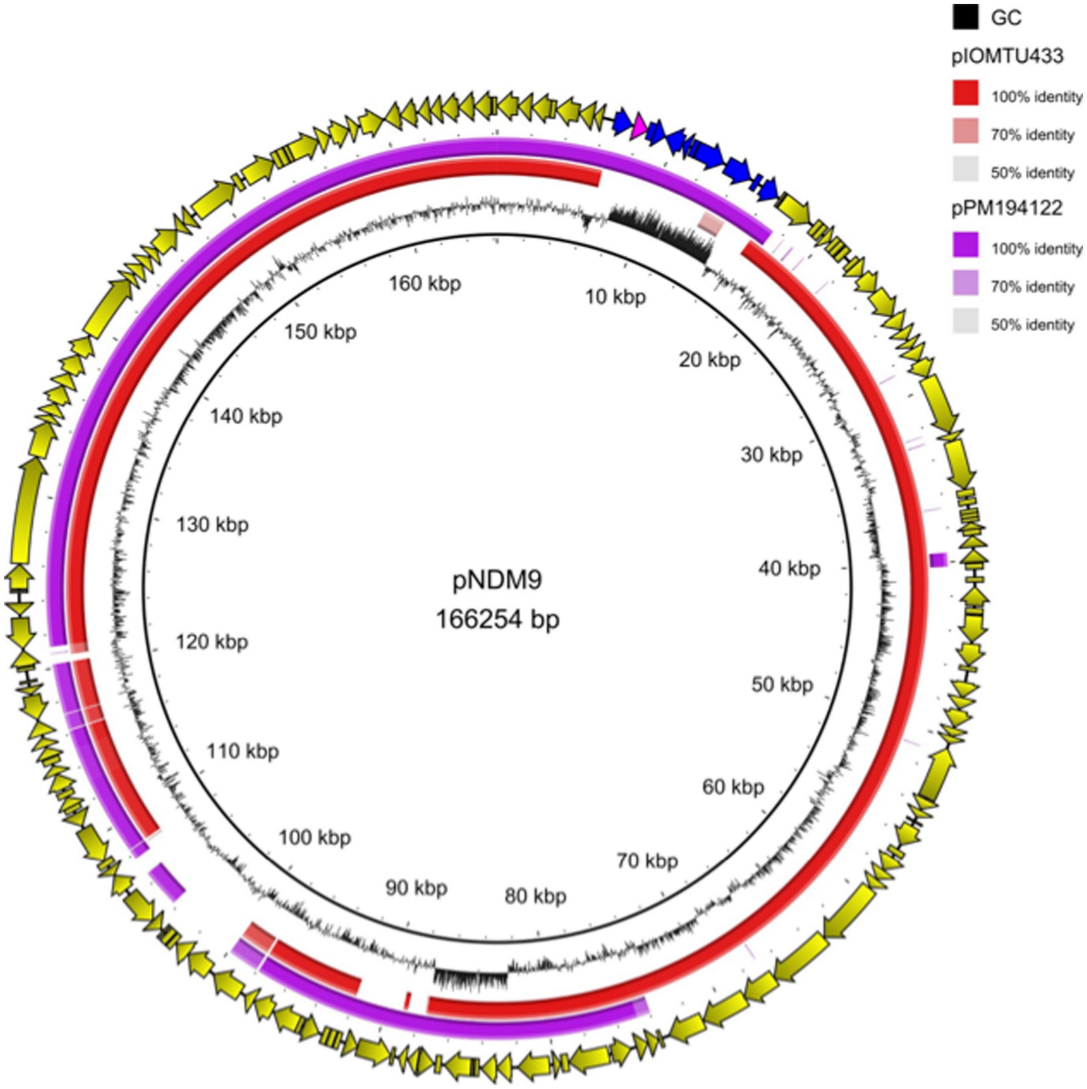
Linear sequence alignment of plasmids pNDM-9, pIOMTU433 (accession number: AP014650) and pPM194122 (accession number: CP050426). Yellow arrows indicate the open reading frames of pNDM-9 plasmid; blue arrows indicate the Tn*125* transposon and pink arrow indicates the *bla*_NDM-9_ gene.

Analysis of Nanopore sequencing data showed that the *bla*_NDM-9_ gene was located on a full-length Tn*125* composite transposon, carried by a 166,254-bp long plasmid named pNDM-9. A 3-bp target site duplication (TTT) identified on both sides of Tn*125* was indicative of a transposition event. Interestingly, the genetic environment of *bla*_NDM-9_ in *A. baumannii* 13A297 corresponded to the original Tn*125* and differed from that identified in *Enterobacterales*. Indeed, IS*26* was associated with *bla*_NDM-9_ in all but one of the plasmids identified in *Enterobacterales*, highlighting that IS*26* may play a key role in the transfer of the *bla*_NDM-9_ gene between *Enterobacterales* (Li et al., 2021). Plasmid pNDM-9 encoded 165 open reading frames and possessed a GC content of 39.1%. It exhibited significant similarity (99.9%) to 75% of the sequence of plasmid pIOMTU433 (accession number: AP014650) (Figure 1). Plasmid pIOMTU433 is a 189,354 bp long plasmid identified in an *A. baumannii* clinical strain isolated in Nepal in 2013. Interestingly, pIOMTU433 did not carry a *bla*_NDM-like_ gene but a *bla*_PER-7_ ESBL-encoding gene. In addition to *bla*_NDM-9_, pNDM-9 also carried other resistance genes, such as *sul2*, *aph(3″)-Ib* and *aph(6)-Id*, which confer resistance to sulfonamides and aminoglycosides, respectively. Furthermore, the pNDM-9 plasmid shared 99.9% nucleotide identity with 90% of the sequence of the *bla*_NDM-1_ positive plasmid pPM194122 (150,385 bp-long, accession number CP050426), which was sequenced from an *A. baumannii* strain from India (Figure 1). Consistent with the identification of an incomplete *tra* operon, attempts to transfer the pNDM-9 plasmid by mating-out assays were unsuccessful.

### 3.3. Antibiotic susceptibilities of transformants with NDM-1 and NDM-9

To clarify the impact of NDM-9 on cefiderocol susceptibility and to compare it to that of NDM-1, both corresponding *bla*_NDM-1_ and *bla*_NDM-9_ genes were cloned and expressed in *E. coli* and *A. baumannii* reference strains. NDM expression from the plasmid pABEC in *E. coli* DH5α conferred resistance to ceftazidime, imipenem, meropenem, and cefiderocol for NDM-9 (8 mg/L) and a decrease in susceptibility to cefiderocol (at least 64-fold) for NDM-1 (2 mg/L), using the pharmacokinetic-pharmacodynamic (PK-PD) cut-off of 2 mg/L (S ≤ 2 mg/L; R > 2 mg/L) (Table 1). Interestingly, DH5α cells transformed with *bla*_NDM-9_-carrying vector pABEC-NDM-9 were 4-fold more resistant to cefiderocol and at least 2-fold to meropenem than those containing pABEC-NDM-1. In agreement with this result, the transfer of the vector pABEC-NDM-9 into the *A. baumannii* CIP70.10 strain led to a higher level of resistance to cefiderocol (MIC >32 mg/L) than that of pABEC-NDM-1 (MIC = 4 mg/L) (Table 1).

### 3.4. Biochemical properties of NDM-1 and NDM-9 against ceftazidime and cefiderocol and molecular modeling

NDM-9 was previously shown to possess slightly increased hydrolytic activity toward meropenem, imipenem and cefotaxime as compared to NDM-1 (Wang et al., 2014). To gain insight into the structural features that may explain the different levels of resistance of NDM-1 and NDM-9 to cefiderocol, both enzymes were purified and steady-state kinetic parameters of ceftazidime and cefiderocol were determined. NDM-9 exhibited a 7-fold higher hydrolytic efficiency (*k*_cat_/*K*_m_) toward ceftazidime, which is linked to a higher turnover rate (*k*_cat_) of this antibiotic by NDM-9 (Table 2). Although weak, a slight hydrolysis of cefiderocol by NDM-1 was evidenced. As compared to NDM-1, NDM-9 possessed a 3-fold higher affinity for cefiderocol, which was associated with a 2-fold increase in its catalytic efficiency (Table 2).

**Table 2:** Steady-state kinetic parameters for hydrolysis of ceftazidime and cefiderocol by NDM-1 and NDM-9.

Substrate	*K*_m_ (μM)		*k*_cat_ (s^−1^)		*k*_cat_/*K*_m_ (μM^−1^. s^−1^)	
	NDM-1	NDM-9	NDM-1	NDM-9	NDM-1	NDM-9
Ceftazidime	37 ± 5	36 ± 3.6	20 ± 1.8	144 ± 17.7	0.55 ± 0.05	3.99 ± 0.5
Cefiderocol	161 ± 14.8	53 ± 3.6	11 ± 1.4	7 ± 0.9	0.069 ± 0.01	0.13 ± 0.008

Data are the mean of three independent experiments. Standard deviations were within 10% of the mean value.

**Figure 2:**
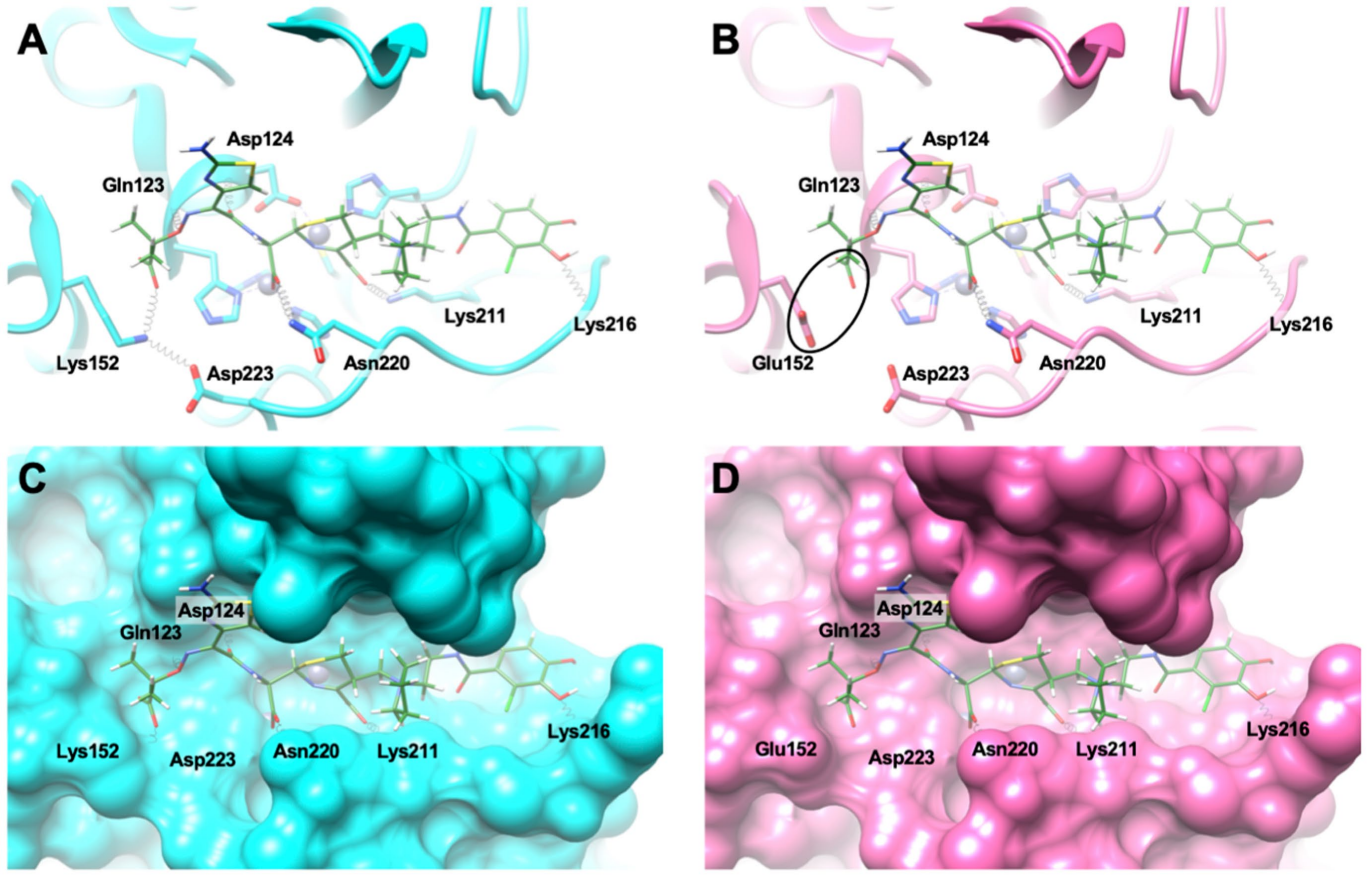
**(A,C)** Docking conformation of cefiderocol (represented as green sticks) in the active site of NDM-9 (represented as cyan cartoon and surface, respectively). **(B,D)** The same docking conformation superposed with the active site of NDM-1 (represented as pink cartoon and surface, respectively). The black ellipse highlights the repulsive interaction between the side chain of Glu152 and the carboxylate group from the R1 substituent in cefiderocol. Hydrogen bonds are represented as black springs.

Molecular modeling revealed stabilizing interactions between cefiderocol and residues Lys211, Lys216, Asn220 of NDM-9, and a strong ionic interaction of Lys152 with both Asp223 and the carboxylate group of R1 substituent in cefiderocol (Figure 2). In NDM-1, this latter favorable interaction is replaced by a repulsive interaction with Glu152, leading to a lower affinity for cefiderocol. Altogether, these results explain the higher cefiderocol MIC and catalytic efficiency observed for NDM-9 as compared to NDM-1.

## 4. Conclusion

To the best of our knowledge, this is the first characterization of an NDM-9 *A. baumannii* clinical isolate, assigned to ST^Pas^25, which belongs to the emerging international clone 7 (IC7). The high level of resistance to cefiderocol of this clinical isolate is at least partly due to the Glu152Lys substitution of NDM-9, but further studies are needed to investigate the contribution of other genetic determinants. The substitution of residue 152 in NDM-9 is unique among NDM variants and it remains to be elucidated whether other substitutions in other NDM variants may have impacts on cefiderocol hydrolysis. Since cefiderocol is the only new active molecule against CRAB strains, the emergence of the NDM-9 variant, which may contribute to high levels of resistance to cefiderocol, is a major threat to human health, given the subsequent therapeutic dead end. As the rates of NDM-positive isolates among carbapenemase-producing *A. baumannii* isolates is increasing in France (1.7% in 2012, 15.9% in 2016 and 32.2% in 2021, French National Reference Centre for Antimicrobial Resistance) and in other countries (Adams et al., 2020; Fam et al., 2020; Kindu et al., 2020; Lukovic et al., 2020) it may seriously compromise the efficacy of cefiderocol for this highly drug resistant pathogen that is among the WHO list of critical bacteria,^2^ for which new antibiotics are urgently needed.

## Data availability statement

The datasets presented in this study can be found in online repositories. The names of the repository/repositories and accession number(s) can be found in the article/supplementary material.

## Author contributions

KJ, RAB, TN, and AP contributed to conception and design of the study. SG, PT, J-BV, SO, and BII performed the experiments. SG, BII, AP, and MB analyzed the data. SG and AP wrote the first draft of the manuscript. AP, PP, KJ, RAB, TN, and BII reviewed and edited the manuscript. All authors read and approved the version.

## Funding

The French National Reference Centre for Antimicrobial Resistance is funded by the French Ministry of Health through the Santé publique France agency.

## Conflict of interest

The authors declare that the research was conducted in the absence of any commercial or financial relationships that could be construed as a potential conflict of interest.

## Publisher’s note

All claims expressed in this article are solely those of the authors and do not necessarily represent those of their affiliated organizations, or those of the publisher, the editors and the reviewers. Any product that may be evaluated in this article, or claim that may be made by its manufacturer, is not guaranteed or endorsed by the publisher.

## Footnotes

^1^ https://pubmlst.org

^2^ https://www.who.int/news/item/27-02-2017s-who-publishes-list-of-bacteria-for-which-new-antibiotics-are-urgently-needed
